# Assessing the effects of adsorptive polymeric resin additions on fungal secondary metabolite chemical diversity

**DOI:** 10.1080/21501203.2014.942406

**Published:** 2014-07-22

**Authors:** Víctor González-Menéndez, Francisco Asensio, Catalina Moreno, Nuria de Pedro, Maria Candida Monteiro, Mercedes de la Cruz, Francisca Vicente, Gerald F. Bills, Fernando Reyes, Olga Genilloud, José R. Tormo

**Affiliations:** ^a^ Fundación MEDINA, Centro de Excelencia en Investigación de Medicamentos Innovadores en Andalucía, Avda. del Conocimiento 3, Parque Tecnológico de Ciencias de la Salud, 18016Granada, Spain

**Keywords:** HPLC-Studio, fungal fermentations, Diaion^®^ resin, Amberlite^®^ resin, secondary metabolites, chemical diversity

## Abstract

Adsorptive polymeric resins have been occasionally described to enhance the production of specific secondary metabolites (SMs) of interest. Methods that induce the expression of new chemical entities in fungal fermentations may lead to the discovery of new bioactive molecules and should be addressed as possible tools for the creation of new microbial chemical libraries for drug lead discovery. Herein, we apply both biological activity and chemical evaluations to assess the use of adsorptive resins as tools for the differential expression of SMs in fungal strain sets. Data automation approaches were applied to ultra high performance liquid chromatography analysis of extracts to evaluate the general influence in generating new chemical entities or in changing the production of specific SMs by fungi grown in the presence of resins and different base media.

## 1. Introduction

Fungal metabolites have historically provided a rich source of lead compounds for drug discovery. However, a general perception is emerging that rates of discovery of new molecules, especially new antibiotics, are decreasing after half a century of continued screening of fungal diversity by fungal fermentation processes (Bills et al. 2009). Recent advances in genome sequencing, facilitated by lowered costs and fast service, have revealed untapped reservoirs of microbial natural products in unexpressed biosynthetic pathways. The number of presumable secondary metabolite (SM) gene clusters in fungal genome analysed so far widely exceeds the number of detected metabolites for these fungal strains (Bergmann et al. 2007; Gross 2007; Hornung et al. 2007). This highlights the opportunity of finding possibly unexpressed genes that potentially could encode the biosynthesis of novel SMs, yet unknown because of our inability to simulate *in vitro* their environmental conditions required for expression (Gross 2007).

The value of empirical strategies for manipulating nutritional and environmental parameters to stimulate the metabolic diversity of a microorganism has long been recognized. In many industrial microbial screening laboratories, empirical comparisons of an organism metabolic response to a large number of medium formulations, over a range of temperatures and aerations, were often the first-line approach for improving product formation and associated bioactivity from a newly discovered fungus or actinomycete. The impact of varied media formulations was also evident during the initial fermentation of large numbers of new strains and testing extracts across multiple biological assays (Yarbrough et al. 1993). Review of screening data often recognized that interesting active metabolites were formed or produced in larger quantities in only one of several media. Recently, several approaches have been applied to foster the expression of these unexpressed pathways and to promote SM biosynthesis. These strategies include the manipulation of medium and growth conditions in miniaturized nutritional arrays (Bills et al. 2008), the application of transformation techniques for the generation of gene knockouts, the exchange of native gene promoters with constitutive or inducible promoters or the overexpression of transcription factors (Brakhage and Schroeckh 2011), the co-cultivation of one or more microorganisms in constant interaction (Combès et al. 2012), the addition of small-molecule elicitors such as epigenetic modifiers, inhibitors of histone deacetylase or DNA methyltransferase and the fermentation in the presence of non-ionic adsorption resins (de la Cruz et al. 2012).

Adsorptive polymeric resins have been incorporated since 1970s into fermentation processes for myxobacteria, fungi and actinomycetes, basically in order to increase titres by sequestering metabolites, preventing degradation or decreasing the cytotoxic effects of the final metabolites produced (Phillips et al. 2013). Several examples of microbial SMs demonstrate that the addition of resins cause some of these effects, including: (a) the production of rubradirin, an antibiotic produced by *Streptomyces achromogenes* subsp*. rubradiris* (Marshall et al. 1990) in which the addition of non-ionic polymeric adsorbents XAD-2, XAD-7, XAD-16, HP-20 and HP-21 led to a two- to fourfold increase in rubradirin titres after 3–4 days of fermentation; (b) paulomycin production, wherein the resin prevented the transformation of paulomycin to paulomenol (Marshall et al. 1987); (c) the production of BMS-182123, a metabolite produced by *Penicillium chrysogenum* which acts as an inhibitor of tumour necrosis factor alpha (TNF-α), whose production was increased by 5.5-fold by adding XAD-8 resin in the fermentation medium (Warr et al. 1996); (d) the production of migrastatin and isomigrastatin, potential anticancer agents produced by *Streptomyces platensis* (Woo et al. 2002), where XAD-16 added to the fermentation medium enhanced its production; (e) the production of ambruticins and jerangolids, new antifungal compounds produced by the myxobacterium *Sorangium cellulosum* (Gerth et al. 1996) and (f) the production of nomimicin, a new spirotetronate class of polyketide obtained from an actinomycete of the *Actinomadura* genus fermented in the presence of the polymeric resin HP-20 (Igarashi et al. 2012).

Our research group also described recently that the production of some metabolites is increased in the presence of non-ionic adsorption resins such as Amberlite^®^ XAD-7 (de la Cruz et al. 2012), sometimes resulting in a modulation of whole-cell antibiotic production. So far, no clear evidence indicated that the addition of non-ionic adsorptive resins to fermentation processes is capable of activating unexpressed biosynthetic pathways, or increasing the chemical diversity produced in fungal fermentations (Phillips et al. 2013). To examine this hypothesis, we have evaluated the effect of different resin additions on fungal SM diversity production using ultra high performance liquid chromatography (uHPLC)-UV profiles and a new data-automated analysis method. Our aim was to generate collections of natural product extracts with high chemical diversity, with the final goal of finding new potential non-cytotoxic antifungal and antibacterial agents in our drug discovery programs.

## 2. Material and methods

### 2.1. Inocula preparation

Cryotubes containing fungal inocula or mycelia discs in 10% (v/v) glycerol stored at −80°C were thawed, and strains were revived and transferred to 60-mm Petri dishes containing 10 ml of YM agar (malt extract 10 g, yeast extract 2 g, agar 20 g, 1000 ml distilled H_2_O) at 22°C for 14–20 days. Five mycelial discs were cut from each 60-mm plate with a sterile Transfer Tube (Spectrum Laboratories Inc., Rancho Dominguez, CA, USA). Mycelia discs were extruded from the Transfer Tube and crushed in the bottom of tubes (25 × 150 mm) containing 12 ml of SMYA medium (Difco™ neopeptone 10 g, maltose 40 g, Difco™ yeast extract 10 g, agar 4 g, distilled H_2_O 1000 ml) and two cover glasses (22 × 22 mm). Tubes were incubated on an orbital shaker (200 rpm; 1.5 cm throw), where rotation of the cover glasses continually sheared hyphae and mycelial disc fragments to produce nearly homogenous hyphal suspensions consisting of minute hyphal aggregates and fine mycelia pellets. Tubes were incubated for 7 days at 22°C (Bills et al. 2008).

### 2.2. Media formulation

MMK2 (mannitol 40 g, Difco™ yeast extract 1 g, Sigma-Aldrich (St. Louis, MO, USA) Murashige & Skoog salt 4.3 g, distilled H_2_O 1000 ml), SCAS (Panreac (Barcelona, Spain) soluble starch from potato 40 g, Sigma-Aldrich casein hydrolysate 5 g, KH_2_PO_4_ 0.5 g, MgSO_4_·7H_2_O 0.5 g, FeSO_4_·7H_2_O 0.01 g and distilled H_2_O 1000 ml), MV8 (maltose 75 g, V8 juice 200 ml, Sigma-Aldrich soy flour 1 g, L-proline 3 g, 2-(N-morpholino)ethanesulfonic acid 16.2 g and distilled H_2_O 800 ml), YES (Difco™ yeast extract 20 g, sucrose 150 g, MgSO_4_·7H_2_O 0.5 g, trace elements 1 ml (ZnSO_4_·7H_2_O 1 g/100 ml and CuSO_4_·5H_2_O 0.5 g/100 ml) and distilled H_2_O 1000 ml), LSFM (glycerol 18.7 g, glucose 40 g, NH_4_SO_4_ 2 g, Sigma-Aldrich yeast autolysate 5 g, soybean flour 5 g, tomato paste 5 g, sodium citrate 2 g and distilled H_2_O 1000 ml) and XPMK (D-Xylose 60 g, Difco™ peptone 8 g, KH_2_PO_4_ 0.5 g, Murashige & Skoog salt 4.3 g, distilled H_2_O 1000 ml). Fermentation vials (42 ml EPA vials) containing 10 ml of medium were autoclaved for 21 min at 121°C.

### 2.3. Addition of adsorptive resins

Four adsorptive polymeric resins were added to fermentations: Amberlite^®^ XAD-2, XAD-7, XAD-16N (hydrophobic polyaromatic resins, Sigma-Aldrich) and Diaion^®^ HP-20 (styrene-divinylbenzene, Supelco (Bellefonte, PA, USA)) polyaromatic adsorbent resin for hydrophobic retention of compounds as antibiotics and other molecules. The resins were washed in twice their volume of methanol and stirred for 1 h, followed by six washes with distilled water, and were finally stored at 4°C in distilled water for 48 h (Singh et al. 2010). Afterwards, they were vacuum filtered through a 125 mm diameter and 6 µm pore size filter. Once filtered, the resins were oven dried at 75°C, and 0.3 g of each resin was added to the fermentation vials before autoclaving for 21 min at 121°C.

### 2.4. Growth

Cultures of each fungus were inoculated by adding 0.3 ml of inoculum suspension into five 42-ml EPA scintillation vials containing 10 ml of culture media with and without each of the resins. The vials were incubated for 14 days in a shaking incubator (Kühner AG, Birsfelden, Suiza) at 22°C, 220 rpm and 70% relative humidity.

### 2.5. Extraction

After 14 days of incubation, fermentation broths with cells were extracted by adding 9 ml of acetone using a Multiprobe II robotic liquid handler and shaking at 220 rpm for 1 h. After centrifugation, 12 ml of supernatant from each vial was transferred to glass tubes containing 0.6 ml of dimethyl sulfoxide (DMSO). Solvent was evaporated under a hot nitrogen stream to a final volume of 3 ml (80/20 water/DMSO solution) and a final concentration of 2× whole broth equivalents. Each fermentation batch included extracts from control culture media to discriminate their components. This extraction procedure was validated using antibiotics covering a wide range of polarities, molecular weights (MWs) and chemical structures, measuring in each case if compounds were detected after the extraction and preparation of the samples. For this purpose, water solutions containing 1 mg/ml of Terramycin^®^, cephalothin, amphotericin B, novobiocin, fusidic acid and actinomycin and unsaturated resins at 3% weight/volume were extracted applying the same methodology.

### 2.6. Bioactivity characterization

The antimicrobial activities of the extracts were evaluated against methicillin-resistant *Staphylococcus aureus* (MRSA) MB5393 (de la Cruz et al. 2012), *Candida albicans* MY1055 (de la Cruz et al. 2012) and *Aspergillus fumigatus* ATCC 46645 (Monteiro et al. 2012). Briefly, the microorganisms were incubated with the extracts for 18–30 h at 37°C. Sample 1:10 or 2:25 dilutions were used depending on the target strain. The activities were measured by monitoring the absorbance differences at 600 nm between the final and the initial incubation times, except for *A. fumigatus* where the activity was scored by using resazurin, an oxidation–reduction indicator of the cell viability (Monteiro et al. 2012). Additionally, the cytotoxicity of the different extracts against the HepG2 cell line (hepatocellular carcinoma, ATCC HB 8065) was evaluated by a classical 3-(4,5-dimethylthiazol-2-yl)-2,5-diphenyltetrazolium bromide reduction colorimetric assay, with the same incubation times and assay concentrations as used for the antibiotic evaluation (de Pedro et al. 2013).

### 2.7. Chemical analysis

Chemical profiles of fermentation extracts were analysed using an Agilent 1290 Infinity uHPLC-diode array detector (DAD). A Kinetex C-18 (1.7 μm, 2.1 × 150 mm) from Phenomenex (Torrance, CA, USA), a 10-min gradient from 1% to 99% (v/v) of acetonitrile in water, with 1.3 mM ammonium formate and 1.3 mM trifluoroacetic acid as chromatographic modifiers, a flow rate of 0.315 ml/min, a controlled temperature of 40°C and UV detection at 210, 280 and 340 nm were used for each analysis. An inert internal plastifier control (IC) was present in each sample to individually validate and normalize if necessary each chromatographic run. Additional methanol blanks were injected every 10 samples for joint monitoring of each analytical batch. Agilent integration uHPLC parameters included tangent skip mode (standard), tail peak skim height ration (0), front peal skim height ration (0), skim valley ratio (0), baseline correction (advanced), peak to valley ratio (1), slope sensitivity (15), peak width (0), area reject (0), height reject (0) and shoulders (TAN).

### 2.8. Data automation

The samples and positions in the storage plates were recorded in a computer database Oracle system (Nautilus LIMS by Thermo) where the producing microorganism, the fermentation medium, the additive, the extraction procedure and the location of each sample in the storage plates were recorded. An improved version of HPLC-Studio (Tormo et al. 2003; Tormo & García 2005) correlated all injected samples and extracts to their corresponding unfermented fermentation broths and allowed the chemical comparisons.

This recent version of HPLC-Studio (García & Tormo 2003; Tormo et al. 2003; Tormo & García 2005), with improvements for its application in uHPLC with Oracle databases (without limitations on the number of traces that could be processed at the same time), was used for data processing. No intensity peak thresholds were used. All the peaks eluting between 1.5 and 9.0 min were processed.

Variations found in the uHPLC retention times in the analysis of complex extracts by HPLC-DAD and uHPLC-DAD were due to several factors, such as the composition of the mixtures, the concentration of the compounds, the existence of molecules with very close retention times and the degree of resolution of the DAD detection and were corrected by applying a window of 0.065 min (double of the DAD scan detecting resolution), previously established as an adequate resolution time to distinguish between two distinct SMs among a large batch of sequential chromatograms (García & Tormo 2003; Tormo et al. 2003; Tormo & García 2005).

### 2.9. Characterization of chemical diversity by profile clustering

Only data related to the presence or absence of newly produced metabolites were used. Peak areas were not taken into account for the analysis to avoid biases due to very large areas and to a possible deficient extraction of all quantities of the produced metabolites. Therefore, interpretations were strictly based on the presence or absence of peaks, without the quantitative data. To validate this approach, extracts of a water solution of several antibiotics (see Section 2.5) were also subjected to uHPLC analysis. Although in some cases the recovery titres were low (especially the one with the lowest polarity, actinomycin; see Supplementary information), the presence of all the antibiotics in the acetone extract confirmed that, in terms of presence or absence of the compounds, the extraction method could be used for chemical comparisons of the microbial SM profiles obtained under different fermentation conditions.

Components of the control fermentation media were subtracted from any SM counting. Chemical characterization of the diversity of the extracts started with the determination of the number of metabolites that were detected in the fermentations versus the control media. Next, the number of different peaks in a single condition versus the number of peaks of the metabolic footprint obtained from all the peaks present among all conditions for a single strain indicated the chemical diversity generated by that strain in each condition (a ratio expressed as a percentage). All fermentation conditions were classified based on their contribution to the chemical diversity following this procedure. The matrix of percentages of overlapping diversity between each condition was also determined.

BioNumerics software version 6.0 (Applied Maths) was used for the comparison of the metabolic footprints of each strain in the different fermentation conditions. The BioNumerics software was loaded with the resulting data matrix generated for the analysis of the common and uncommon peaks generated by the software (García & Tormo 2003; Tormo et al. 2003; Tormo & García 2005). The statistical analysis in BioNumerics was based on the determination of the Dice similarity coefficient (Dice 1945). From the estimated similarity coefficients, similarity dendrograms were generated by the unweighted pair group with arithmetic averages (UPGMA) method. This method uses the arithmetic average to generate clusters, showing the similarities between the different fermentation conditions based on the presence or absence of common SMs in each case. Principal component analysis (PCA) was also calculated with the BioNumerics software to facilitate visualization of relationships among the different fermentation conditions per strain.

### 2.10. Chemical profiling validation for adsorptive resins comparisons

Strategies for automated evaluation on SM profiles with automated HPLC peak database comparison have been outlined previously by our group to select the most chemically diverse condition before scale-up (García & Tormo 2003; Tormo et al. 2003; Tormo & García 2005). The same methods could be used statistically to assess the real influence on the metabolic profiles caused by the adsorptive polymeric resin additions. However, the degree of sensitivity and accuracy of the method had to be validated to confirm that the degree of variation observed within triplicates was always smaller than the one observed among the different fermentation conditions evaluated for the resins.

For this purpose, three different species of fungi were selected and fermented in triplicate, in two different base media each, with three different fermentation conditions (control media, control media plus XAD-16 and control media plus HP-20 additives). Chemical profile results proved that the previous method developed for the comparison of different metabolic profiles (presence or absence of detected metabolites by uHPLC at 210 nm and further statistical analysis by Dice + UPGMA from uHPLC-Studio + BioNumerics; Tormo & García 2005) was sufficiently sensitive that the triplicates had narrower distance coefficients than the statistical distances observed among the varied fermentation conditions.

## 3. Results and discussion

### 3.1. Bioactivity evaluation of sets of strains

**Figure 1.**
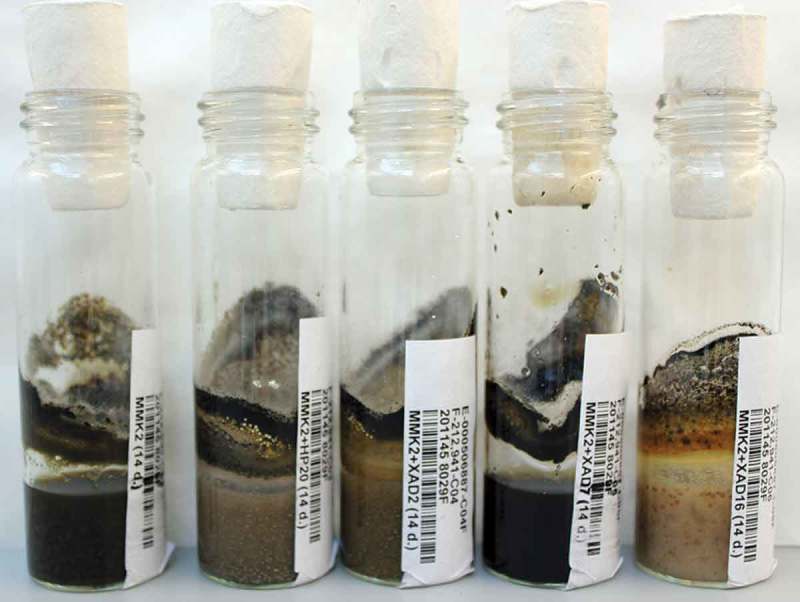
Fungal strain CF-212941 grown on MMK2, MMK2+HP-20, MMK2+XAD-2, MMK2+XAD-7 and MMK2+XAD-16 at 14 days, showing differences in texture, colour, morphology and quantity of biomass.

Ninety-six different species of filamentous fungi from the Fundación MEDINA strains collection, belonging to orders known to be typical sources of biologically active molecules, were selected: *Chaetothyriales, Diaporthales, Dothideales, Eurotiales, Helotiales, Hypocreales* and *Pleosporales*. After 14 days of fermentation in MMK2 medium in the presence or absence of resins, differences in texture, colour, morphology and biomass were observed among the different resins when harvested (Figure 1).

**Figure 2.**
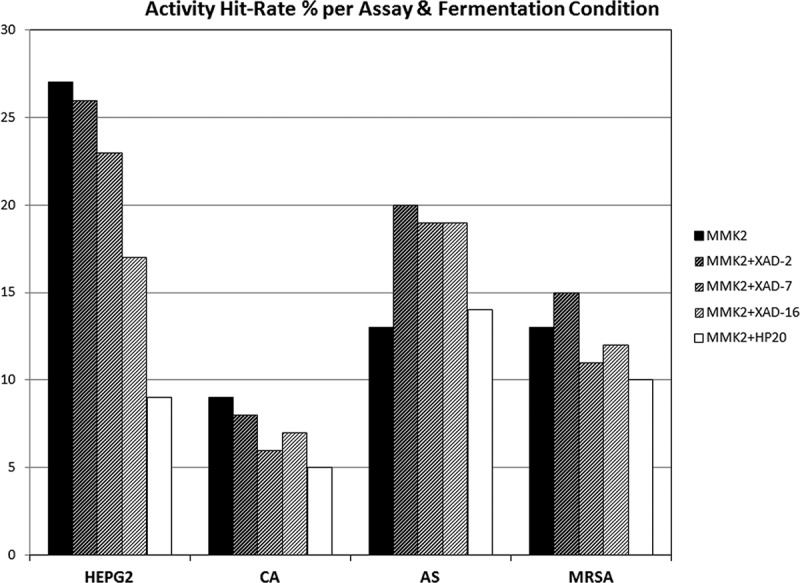
Activity hit-rate (%) of extracts, per assay and fermentation condition for the 96 fungal strains tested. HEPG2, cytotoxicity assay in HepG2 cell line. CA (*Candida albicans*), AS (*Aspergillus fumigatus*) and MRSA (methicillin-resistant *Staphylococcus aureus*) anti-infective tests.

Assuming that the phenotypic difference could indicate different SM production and therefore different potential new chemical entities, we proceeded to their extraction and evaluation for anti-infective and cytotoxic properties against three relevant human pathogens: one Gram-positive bacterium, MRSA and two fungi (*Candida albicans* and *Aspergillus fumigatus*). Since many antibacterial and antifungal metabolites also exhibit human cell cytotoxicity, this biological activity was also added to the assay panel (Figure 2).

Results indicated that, in general, for the MMK2 base fermentation medium, the addition of Diaion^®^ HP-20 resin resulted in a decrease in cytotoxic and antimicrobial active hit-rates, whereas the addition of Amberlite^®^ XAD-2 showed generally higher antimicrobial and cytotoxicity activity hit-rates than XAD-16 or XAD-7, which presented intermediate activity hit-rates.

Because most of the strains did not differ significantly in their activity profiles whether fermented in the presence or absence of the resins, we therefore focused further detailed studies on the subset of strains that exhibited consistent changes of their activity profiles when fermented in the presence of the resins, as a clear indicator of the existence of real changes in their metabolite profiles, including changes in metabolite concentrations. For this purpose, 14 strains from the original 96 were selected for chemical analysis because they presented consistent clear variation patterns on their activity profiles among the different target strains (Table 1). This subset of strains covered a wide taxonomic space for assessing the effect of the addition of the resins on a wide variety of fungi, including orders: Eurotiales (*Penicillium*), Hypocreales (*Cordyceps, Bionectria, Fusarium, Cosmospora*), Pleosporales (*Preussia, Phoma, Sydowiella*), Chaetothyriales and Diaporthales (*Pilidiella, Diaporthe*).

**Table 1.** Antibiotic activities of the fungal strains selected.

Strain ID	TAXINIT	Country	HEPG2	CA	AS	MRSA
CF-194989	*Phoma* sp.	Puerto Rico	+++++	−+−−−	+++++	−+−−−
CF-195017	*Preussia* sp.	Puerto Rico	−+−−−	−−−−−	−+−−−	−+−−−
CF-209155	*Preussia intermedia*	Portugal	+−−−−	−−−−−	−−−−−	−−−++
CF-209171	*Preussia* sp.	South Africa	−+−+−	−+−+−	−+−+−	−+−+−
CF-209591	*Cordyceps* sp.	New Zealand	−++−−	−++++	−+++−	+++−−
CF-210345	*Sydowiella* sp.	Puerto Rico	−−−−−	−−−+−	−−−+−	−++++
CF-210367	*Cosmospora vilior*	Spain	+−−−−	−−−−−	+−−−−	+−−−−
CF-210370	*Bionectria* sp.	Spain	+−−−−	−−−−−	−−−−−	+−−−−
CF-210988	*Penicillium* sp.	C. African Republic	−−−+−	−−−−−	−++++	−−−+−
CF-210989	*Fusarium* sp.	C. African Republic	−−−+−	−−−−−	−−−−−	−+−−−
CF-214546	*Diaporthe* sp.	C. African Republic	−+−−−	−−−−−	−−−−−	−+−−+
CF-214552	*Pilidiella castaneicola*	New Zealand	+−−−	+−−−−	−−−−−	+−−−−
CF-214558	*Penicillium* sp.	C. African Republic	−−−+−	−−−−−	−−−−−	−+−−−−
CF-214575	*Chaetothyriales* sp.	Spain	−−−−	−++++	−++++	−−−−−

Notes: HEPG2: hepatocellular carcinoma ATCC HB 8065; CA: *Candida albicans* MY1055; AS: *Aspergillus fumigatus* ATCC 46645; MRSA: methicillin-resistant *Staphylococcus aureus* MB5393. Sequentially: MMK2 (±), MMK2+XAD2 (±), MMK2+XAD7 (±), MMK2+XAD16 (±) and MMK2+HP-20 (±).

Among all the fermentation conditions tested, XAD-16 and HP-20 resins were also initially selected for further detailed studies because they were associated with larger differences in activity hit-rates versus base medium conditions (Figure 2). According to the manufacturers, both resins have similar chemical properties (styrene-divinylbenzene): XAD-16 is mostly used for adsorption of organic compounds of medium MWs from aqueous systems and polar solvents and HP-20 is used for refining of pharmaceuticals and natural extracts because it adsorbs larger molecules due to its relatively large pore sizes. XAD-16 characteristics included 560–710 µm of particle size, 200 Å of pore size and 800 m^2^/g of surface area, whereas HP-20 characteristics included 250–850 µm of particle size, 260 Å of pore size and 500 m^2^/g of surface area. The larger particle surface and slightly smaller pore size, but higher number of pores, make XAD-16 more suitable for hydrophobic compounds up to MW of 40,000, especially proteins. On the contrary, HP-20, with slightly larger pore sizes, can also be used for larger MW compounds.

### 3.2. Effect of resins on the fungal growth

Furthermore, in order to determine whether the changes in SM obtained by the fermentation in presence of the resin additives were due to changes in the initial composition of the medium (i.e. by having the resin capturing essential components prior to the inoculation), we pre-treated the MMK2 control media with the resins prior to their filtration and autoclaving. The sucrose-rich medium (YES) was also tested to evaluate if possible differences could be medium dependent. YES medium was selected because of its historical success for enhancing the production of active SMs (Bills et al. 2008).

**Figure 3.**
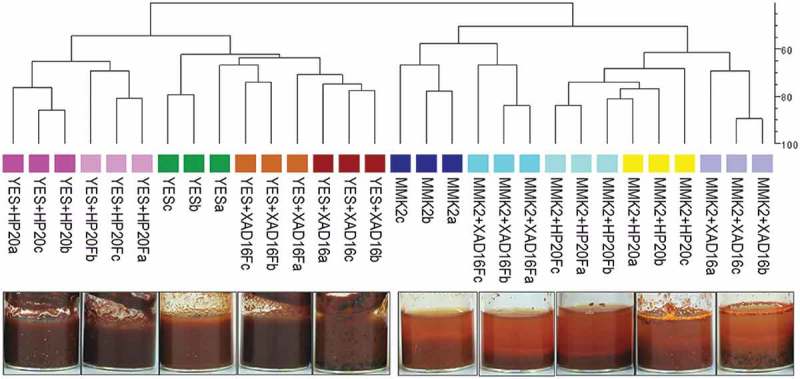
UPGMA dendrogram showing overall similarities between SMs produced by fungal strain (CF-210988) when grown on 10 different fermentation conditions per triplicate, comparing the different fermentation triplicates (a, b, c), the different resins (HP-20 or XAD-16) and two base media autoclaved in the presence of the resins or just pre-treated before inoculating the fungal strain (Fa, Fb, Fc). Similarities were determined by Dice’s coefficient values.

In general, the addition of the resins to the fermentations caused variations in the morphology, colour and amount of biomass in many of the broths, with fermentation triplicates behaving homogeneously. For example, in *Penicillium* sp. strain CF-210988 grown in YES medium (Figure 3), the treated and pre-treated conditions with XAD-16 or HP-20 produced more biomass than the control medium, with no substantial chemical differences between pre-treated and treated media. Interestingly, colour from either pre-treated or treated conditions was equally different when compared to the control condition, indicating that the pre-treatment with the resins had affected the growth to a certain extent. These effects might be explained by a potential sequestration by the resin of trace elements or some components of the medium during the pre-treatment.

However, in the case of the MMK2 medium, the XAD-16 pre-treated condition was very similar to the control medium, whereas the HP-20 pre-treated condition was more similar to both XAD-16- and HP-20-treated media. When the same strain was grown in MMK2 medium, only the XAD-16-treated medium exhibited increased biomass production and different chemical profile compared to the control. Interestingly, for both media, the HP-20 resin surface remained white after harvesting, whereas XAD-16 resin became strongly pigmented in both media (Figure 3).

In general, differential production profile and more biomass quantities were always observed when treatments with resins were performed along the whole fermentation processes. This observation and the literature references to the production of certain metabolites related to product displacement mechanisms along the whole fermentation process determined us to focus our further chemical profiling studies only on full-treated fermentation conditions.

### 3.3. Chemical evaluation of the resin addition to fungal fermentations

**Figure 4.**
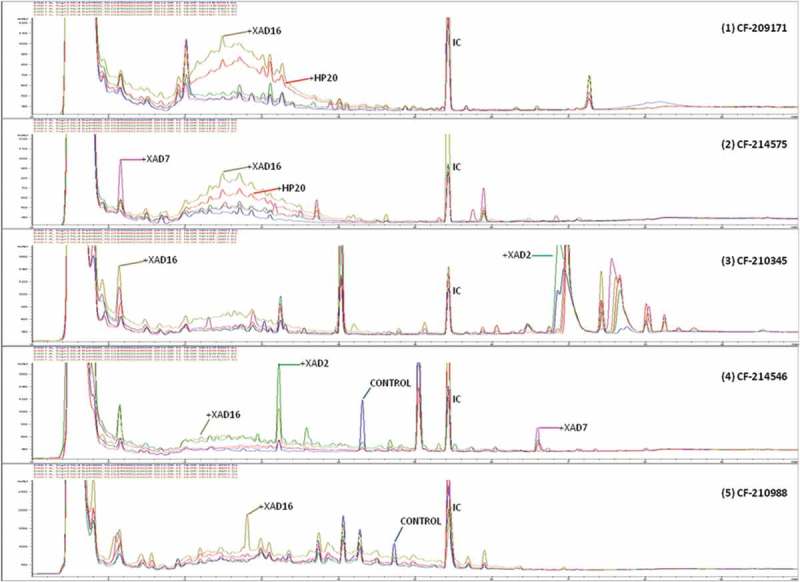
Comparative analysis of different uHPLC-UV 210 nm secondary metabolite profiles produced by five different fungal strains: (1) *Preussia* sp. (CF-209171); (2) *Chaetothyriales* sp. (CF-214575); (3) *Sydowiella* sp. (CF-210345); (4) *Diaporthe* sp. (CF-214546); (5) *Penicillium* sp. (CF-210988) when these fungus were grown on MMK2 fermentation medium with and without XAD-2, XAD-7, XAD-16 and HP-20 resins. Relevant uHPLC traces are indicated in the figure. Internal control (IC) was added homogeneously to each sample to allow accurate comparisons of the chromatographic runs.

To characterize in detail the differences observed within the set of 14 fungal strains with or without adding the resins during the whole fermentation process, we decided to compare their SM profiles by uHPLC. Analysis of their SM chemical profiles (Figure 4) proved that for each of these strains, there could be: (i) conditions with clear improvement in the quantity of production of given compounds in the presence of the resins, (ii) compounds that only were produced in the control medium (without resins) and (iii) compounds that were only present when specific resins were added. The large number of cases found with variations in the presence or absence of specific SMs, determined by counting the variations in the number of compounds detected by uHPLC, led us to a practical representation on the real effect of the resins for this set of fungal strains.

**Figure 5.**
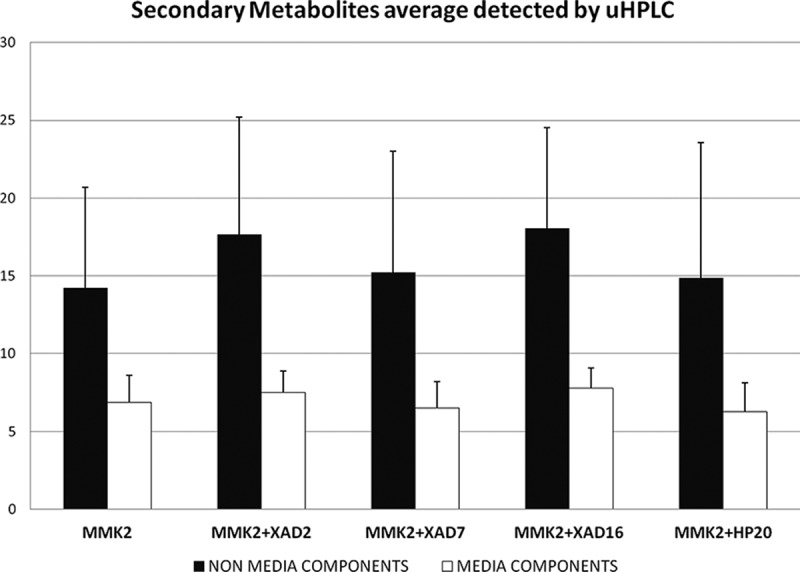
Average number of secondary metabolites detected by uHPLC-DAD for each additive to the MMK2 control medium fermentation for the 14 fungal strains that responded positively to the resin addition. The *T*-test method was used to compare pairs; pair differences were not statistically significant at *α* = 0.05.

**Figure 6.**
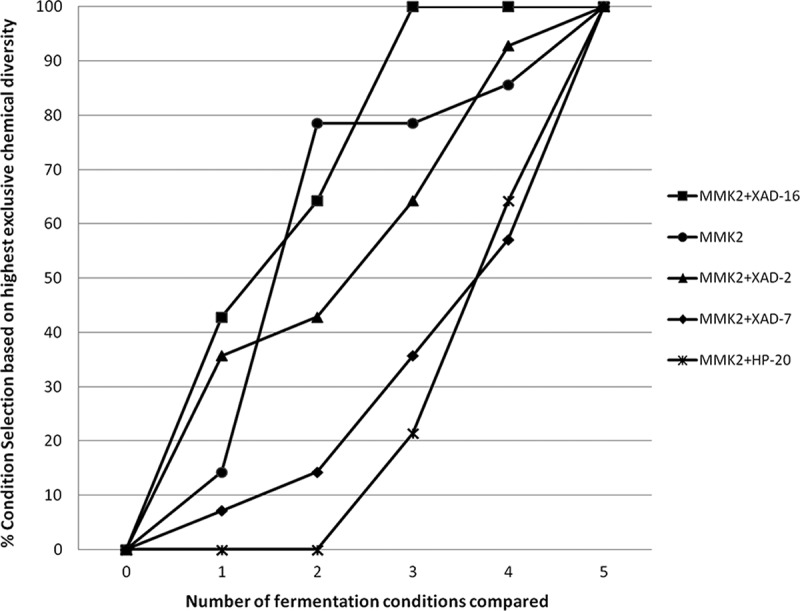
Percentage of fermentation conditions that presented highest exclusive chemical diversity as a function of number of fermentation conditions compared to evaluate the contribution to greater chemical diversity detected by uHPLC for five different fermentation conditions tested in 14 fungal strains that responded positively to the resin addition.

Initially, the difference in the number of SMs produced by each strain with added resins was not significant for any of the four additives tested in the MMK2 medium (Figure 5). However, to determine the best condition for enhanced chemical diversity production, we scored the frequencies of the conditions that generated the maximum non-overlapping chemical diversity for each strain versus the total number of conditions used per strain. An averaged scoring was obtained for the representative subset of 14 strains (Table1, Figure 6). According to this methodology (García & Tormo 2003; Tormo et al. 2003; Tormo & García 2005), displacements of a condition from the 1:1 diagonal linear correlation clearly highlight different chemical diversity influences: (i) the greater the displacements towards the upper part of the graph, the better the medium results for exclusive new SM generation; whereas (ii) displacements towards the lower half of the graph indicate lesser differential metabolite generation (Figure 6).

This analysis clearly indicated that the order of influence of the resins in inducing more to less chemical diversity was sequentially XAD-16 followed by XAD-2, XAD-7 and HP-20. This result could be correlated, to a certain extent, with the low bioactivity hit-rates observed in the anti-infective and cytotoxic assays for the HP-20 resin (Figure 2), which in turn, could be related to the reduction in the chemical diversity also observed of these fermentations.

To determine whether these effects were dependent or not on the composition of the basal medium and, whether changes in carbon, nitrogen or trace elements sources could have greater influences on the production of SM than simply the presence of the resins, the same 14 fungi were also fermented in other five media with completely different compositions in carbon, nitrogen and trace elements sources (LSFM, MV8, SCAS, XPMK and YES) (Bills et al. 2008), with and without XAD-16 and HP-20 resins.

**Figure 7.**
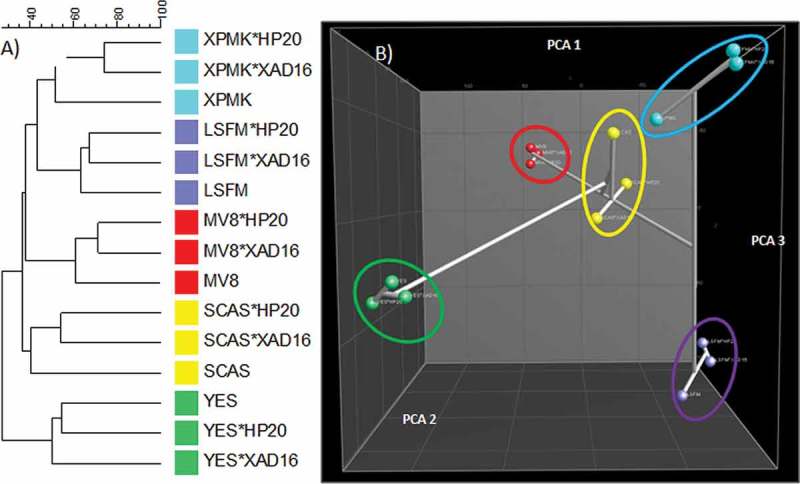
(A) DICE-UPGMA dendrogram. (B) Principal component analysis (PCA) of the secondary metabolites produced by the 14 selected strains from the study that responded positively to the resin addition grown on 15 fermentation conditions. PCA three-dimensional representation only depicts the three principal statistical components.

In general, and in agreement to the MMK2 results (Figure 5), the number of SMs produced did not differ significantly in any of the conditions (results not shown). Dice + UPGMA comparison of the chemical profiles for all 14 strains confirmed that the adsorptive polymeric resins generated slightly lower modifications in the SM profiles than changes in the composition of the base media (Figure 7A). This could also be observed by other statistical methods when principal components were calculated for this set of strains (Figure 7B). Clear grouping of all conditions belonging to each separate medium indicated that more chemical diversity was achieved by changing the medium composition than by adding the Diaion^®^ or Amberlite^®^ resins.

**Figure 8.**
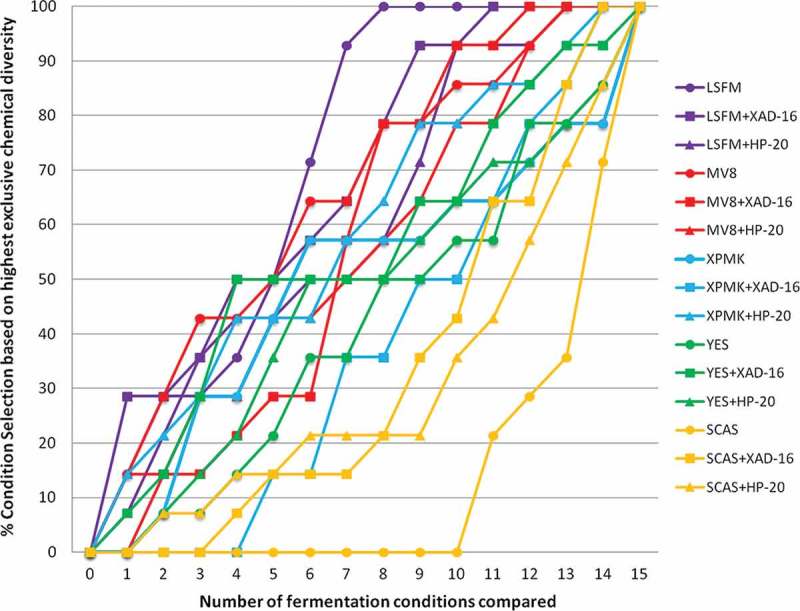
Ranking in percentage of the fermentation conditions that presented the highest exclusive chemical diversity as a function of number of all fermentation conditions compared per strain. To evaluate the contribution to greater chemical diversity detected by uHPLC, 15 different fermentation conditions were tested for the 14 selected fungal strains that responded positively to the resin addition.

Specifically, the medium that generated a higher range of chemical diversity was LSFM followed by MV8, XPMK, YES and SCAS (Figure 8). This analysis also confirmed that changes in the composition of the fermentation medium determined higher variations in the chemical profiles than the addition of the resins. However, the detailed scoring of each condition (LSFM > LSFM+XAD-16 > LSFM+HP-20; MV8+XAD-16 > MV8 > MV8+HP-20, XPMK+HP-20 > XPMK > XPMK+XAD-16, YES+XAD-16 > YES+HP-20 > YES and SCAS+XAD-16 > SCAS+HP-20 > SCAS) indicated that, for fermentation media where a large chemical diversity production is initially observed, such as in LSFM, MV8 and MMK2, the fermentation media without resins generated similar or higher diversity than the one obtained by the addition of XAD-16 or HP-20. However, for fermentation media that induced lower metabolite diversity production, such as SCAS and YES, the addition of XAD-16 or HP-20 resulted in a clear improvement of the chemical diversity profiles generated (Figure 8).

### 3.4. Unique secondary metabolites and changes in production obtained by resin addition

**Figure 9.**
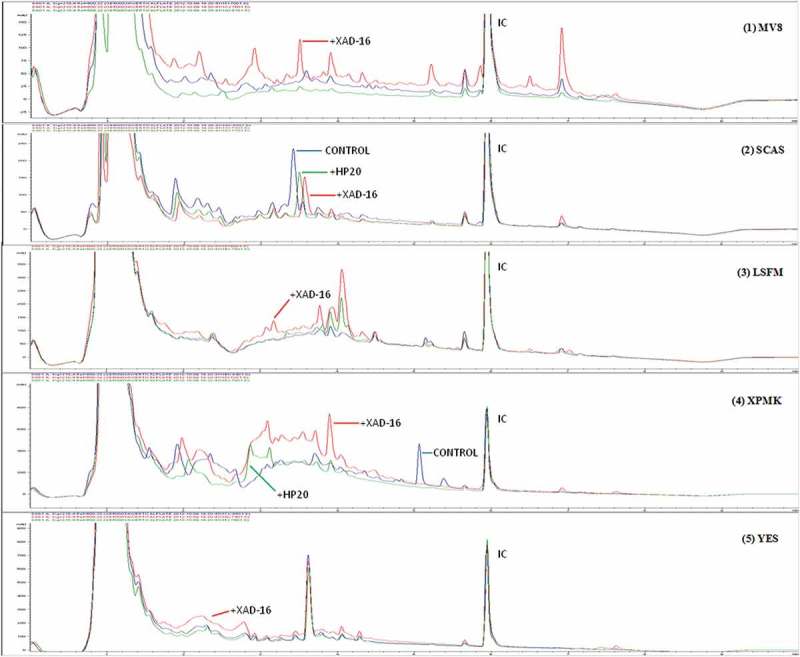
Comparative analysis of different uHPLC-UV 210 nm secondary metabolite profiles produced by strain *Preussia* sp. (CF-209171) when this fungus was grown on five different fermentation media (MV8, SCAS, LSFM, XPMK and YES) with and without XAD-16 and HP-20 resins. Relevant uHPLC traces are indicated in the figure. Internal control (IC) was added to each sample to allow accurate comparisons of the chromatographic runs.

During the statistical characterization of the chemical profiles generated with the addition of the resins, it became apparent that most of the fermentation conditions presented unique SMs and differential production titres of specific compounds. Figure 9 depicts an example (*Preussia* sp. CF-209171) where only MV8, SCAS and XMPK+PH-20 extracts resulted in inhibition zones against *Candia albicans*. In this strain, unique uHPLC peaks could be detected in 66% of the conditions: MV8, MV8+XAD-16, SCAS, SCAS+HP-20, SCAS+XAD-16, LSFM, LSFM+XAD-16, XPMK, XPMK+XAD-16 and XPMK+HP-20, and increments in the quantity of certain metabolites with respect to the control media could be detected in 50% of the resin conditions: MV8+XAD-16, SCAS+XAD-16, LSFM+XAD-16, XMPK+XAD-16 and YES+XAD-16.

**Figure 10.**
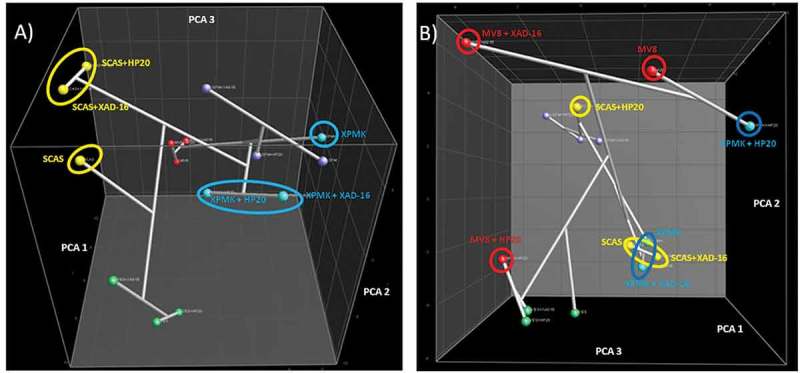
Principal component analysis (PCA) of the total of secondary metabolites produced by (A) *Pilidiella castaneicola* (CF-214558) and (B) *Preussia* sp. (CF-209171) grown in 15 different fermentation conditions. Relevant fermentation conditions are indicated in the figure.

Scoring on the number of unique metabolites per condition in the set of 14 strains supported this hypothesis (Table 2) where 102 unique metabolites were only obtained with the addition of the resins. These numbers clearly indicated that the real value of the addition of the resins to fungal fermentation on large sets of strains really consists in a general increment of the chemical diversity as an overall feature. However, the cause of these improvements remains unknown; they could be due to increased production beyond the detection threshold due to relief from feedback inhibition, relief from auto-toxicity, prevention of end product degradation, change on media composition by adsorption of certain components on the resins or other mechanisms. Moreover, and for some specific cases, the addition of the adsorptive polymeric resins could result even in larger changes in the SM profiles than the ones obtained by changing the composition of the base medium (Figure 10). These results clearly support the application of the adsorptive polymeric Amberlite^®^ or Diaion^®^ resins for specific strains where an increase in the production of specific metabolites or the generation of new bioactive metabolite cannot be achieved solely by introducing major changes in the medium components, e.g. addition of complex plant-based nutrients, or varying the carbon, nitrogen sources and the trace elements.

**Table 2.** Number of unique secondary metabolites detected per microorganism and fermentation condition.

Strain ID	LSFM	LSFM+HP-20	LSFM+XAD-16	MV8	MV8+HP-20	MV8+XAD-16	SCAS	SCAS+HP-20	SCAS+XAD-16	XPMK	XPMK+HP-20	XPMK+XAD-16	YES	YES+HP-20	YES+XAD-16
CF-194989				*5*	*4*				*1*		*1*		*1*		
CF-195017	*1*	*1*	*2*						*3*		*1*			*1*	
CF-209155	*1*		*1*	*2*	*1*			*1*	*2*	*1*				*1*	*2*
CF-209171	*3*		*1*	*2*		*5*	*1*	*1*	*1*	*1*	*1*	*1*			
CF-209591	*2*		*2*	*3*		*6*		*1*	*4*	*1*	*1*	*1*	*1*		
CF-210345	*1*	*2*	*2*	*2*	*1*	*1*			*1*		*1*		*1*		*1*
CF-210367	*2*		*1*		*2*	*1*							*1*	*2*	
CF-210370						*1*	*1*			*1*					
CF-210988		*1*		*3*						*2*		*1*		*1*	*1*
CF-210989	*1*		*3*					*2*	*1*		*1*		*1*	*1*	*1*
CF-214546		*1*			*1*	*1*								*3*	*2*
CF-214552			*1*	*3*		*1*	*1*		*3*	*2*			*2*	*1*	
CF-214558	*1*		*1*			*1*	*1*		*3*		*1*		*1*		
CF-214575	*1*	*1*	*2*	*1*					*1*	*1*			*3*		*2*
Accumulated	*13*	*6*	*16*	*21*	*9*	*17*	*4*	*5*	*20*	*9*	*7*	*3*	*11*	*10*	*9*

## 4. Conclusions

Several studies, on bacteria and fungi, have described the ability of adsorptive polymeric resins to increase the production titres of certain compounds of microbial origin (Frykman et al. 2006), and the possible mechanisms of action for these have been reviewed (Phillips et al. 2013). However, the question of whether one resin is consistently better than others for increasing metabolite chemical diversity has not been examined for any sets of strains. In order to evaluate this possibility, we selected the four most common resins used as microbial fermentation additives – Amberlite^®^ XAD-2, XAD-7 and XAD-16, and Diaion^®^ HP-20 – and we tested them in a pilot study for a set of 96 diverse fungal strains, grown in MMK2 fermentation medium, looking for increasing hit-rates of anti-infective activity and enhancements on metabolite production.

We were unable to correlate the additions of Amberlite^®^ or Diaion^®^ resins to the fermentation media of any fungal species with a direct increase in the production of non-cytotoxic antimicrobial metabolites for the widely diverse set of 96 fungal strains tested. Our bioactivity evaluation tests indicated that the addition of Diaion^®^ HP-20 resin to the base fermentations medium MMK2 resulted in a decrease in the generation of cytotoxic and antimicrobial activities for these strains, whereas their fermentation in MMK2 medium, in the presence of Amberlite^®^ XAD-2, slightly increased the frequency of detection of antibiotic and cytotoxic activity.

Analyses of their SM profiles indicated that variations in the composition of the base media increased the chemical diversity generated by the fungal strains to a larger extent than the addition of Amberlite^®^ or Diaion^®^ resins. It was also observed that variations on the end products of fungal fermentations in the presence of these polymeric resins could be related to an initial capture of essential nutrients by the resins or to a continuous action of the resin throughout the whole fermentation process depending on the base medium composition.

Most of the fermentation conditions tested, either base medium changes, or additions of Amberlite^®^ or Diaion^®^ resins, produced unique compounds not found in any of the other conditions or induced changes in the production of specific compounds in most of the strains. Moreover, for certain strains, the addition of the adsorptive polymeric resins can result in greater changes in the SM profiles than changes obtained solely by varying the carbon, nitrogen and trace element composition. These findings might justify testing the addition of these resins when the production of a specific metabolite encoded by the fungal genome is desired.

In summary, this study is the first data-automated analysis on the effect of the addition of Diaion^®^ and Amberlite^®^ resins to fungal fermentations and their chemical diversity. Although changes in the medium composition more strongly influenced the SMs produced than the addition of adsorptive resins, the results clearly supported the hypothesis that the application of resins in fungal fermentations can determine the production of new SMs and provide a tool to consistently increase the production of low titre compounds. We believe these methods can be generally applied for the generation of natural product extract collections to get access to new bioactive chemical entities in drug discovery programs.
